# Family engagement in care for medical trainees and early career clinicians

**DOI:** 10.1186/s12909-023-04792-2

**Published:** 2023-10-27

**Authors:** Joshua Solomon, Michael Goldfarb

**Affiliations:** 1 Faculty of Medicine and Health Sciences, McGill University, Montreal, QC Canada; 2 Azrieli Heart Centre, Division of Cardiology, Jewish General Hospital, McGill University, 3755 Cote Ste Catherine Road, Office E-212, H3T 1E2 Montreal, QC Canada

**Keywords:** Medical education, Medical training, Family engagement, Clinical care

## Abstract

Engaging family members in care improves person- and family-centered outcomes. Many healthcare professionals have limited awareness of the role and potential benefit of family engagement in care. This review describes the rationale for engaging families in care, and opportunities to engage family in various clinical care settings during training and early career practice.

## Introduction

Family engagement in health care involves a paradigm shift in care delivery. In a family engagement approach, family members are considered partners in care and health care providers support active family involvement in care. Due to societal trends, family members increasingly wish and expect to be more involved in their relative’s care. “Family” in this context is defined broadly and may refer to a relative, caregiver, or close friend.

Engaging family in care involves a collaborative effort between family, the health care team, and the health care system. However, the clinician’s role in supporting family engagement has not been well-defined. Many clinicians have limited familiarity with the potential role and benefits of family engagement, as well as the opportunities to facilitate and promote family engagement throughout the care trajectory. Medical trainees and early career professionals are at opportune stages to learn about and incorporate family engagement principles into routine practice.

This article will describe the rationale for engaging families in care, highlight current curricular competencies for trainees, and outline opportunities to engage family in various clinical care settings during training and early career practice.

## Rationale to engage families in care

An acute care hospitalization can be a very challenging physical, mental, and emotional experience for both patients and family members. The psychological impact that can occur during hospitalization may cause depressive and anxiety symptoms and lead to post-traumatic stress following discharge for patients and their family [1]. Engaging families in care during acute illness has been shown to improve person- and family-centered outcomes, such as improved patient experience, medical goal achievement, family satisfaction, and family mental health outcomes [2, 3]. Family with their needs met are less likely to experience physical and mental stress and are more capable of offering support to their relative [4].

Engaging patients and families in care may also be beneficial for the clinician as well. Many of the engagement techniques build the care relationship and this may provide the clinician with a greater sense of meaning and fulfilment. Family-centered care can also improve the learning environment for medical trainees and increase satisfaction with work life [5].

## Opportunities to engage families in care

**Fig. 1:**
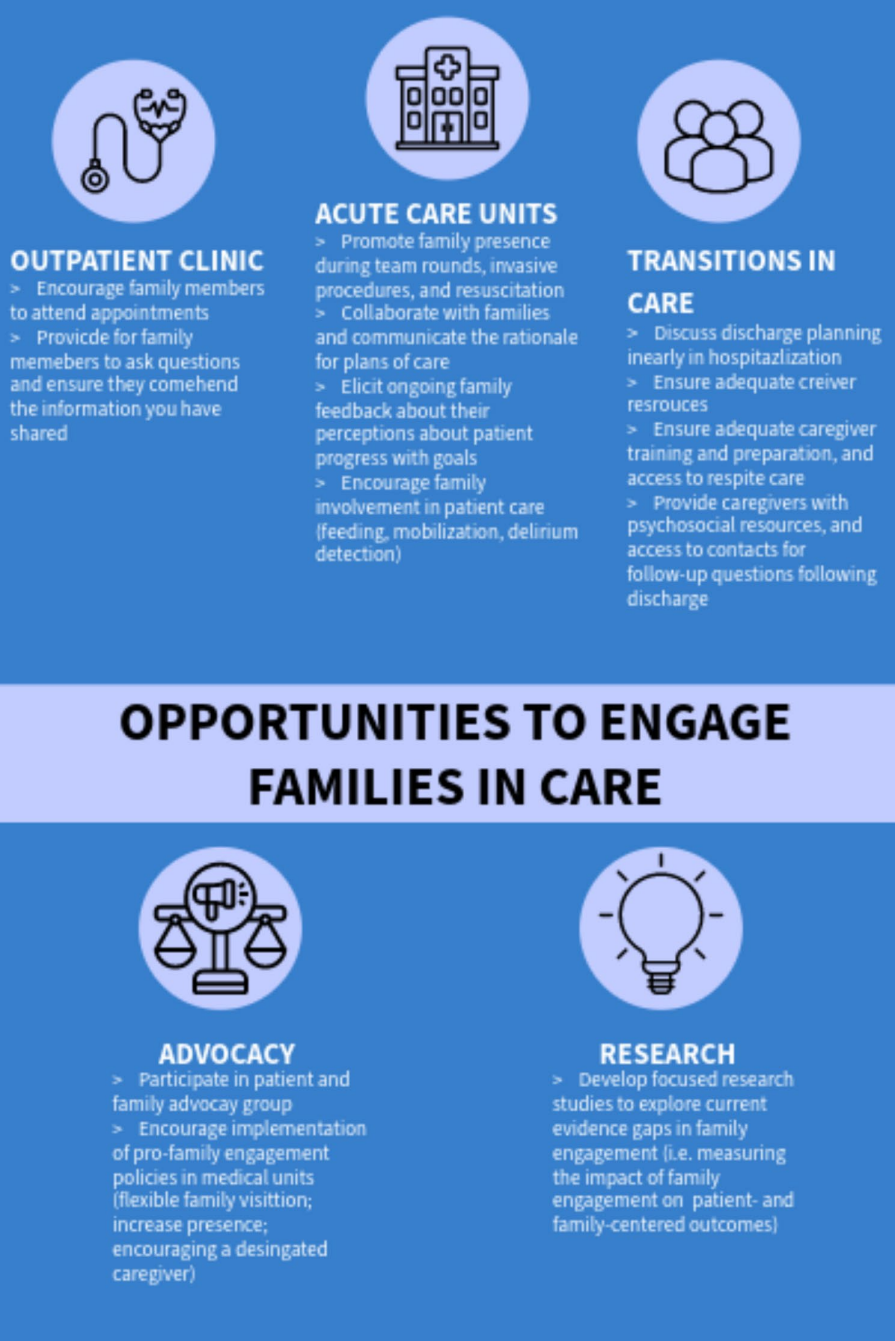
Opportunities for trainees and early career clinicians to engage family in care

Trainees and early career clinicians encounter many situations where they can engage with family members (Fig. 1). In the acute care setting, family can be invited into the room during invasive procedures and resuscitation efforts. Family presence has been shown to have a positive impact on family members and does not appear to affect the procedure [6]. Family can be permitted to observe bedside interdisciplinary team rounds or, when appropriate, even be encouraged to actively participate in the discussion. Trainees can be proactive in having goals of care discussions with family early during hospital stay, which is associated with improved family outcomes and reduced length of hospital stay [2]. Frequent, short communication between clinicians and family using a structured communication tool, either in-person or virtually, can ensure that key information is conveyed and family concerns are addressed while being respectful of limitations in clinician time. Family can be encouraged to contribute directly to certain care activities, such as feeding, mobilization, and delirium detection. Family involvement in mobilization activities has been shown to help patients regain their autonomy and makes family members feel empowered [7]. Family can also play an integral part of the shared decision-making process, either as a surrogate decision maker to ensure that the plan reflects their relative’s expressed beliefs, preferences, and values, or to support their relative’s care decisions. Vulnerable groups, such as people with cognitive disabilities, language barriers, lower health literacy, as well as younger children and older adults, may particularly benefit from having family members actively involved in care. During times when there are severe restrictions in visitation (i.e., during a pandemic), a single designated family member could be encouraged to be present during hospitalization. Family physical health needs can also be assessed at the time of their relative’s hospitalization. For example, one study looking at referral of family members of people hospitalized with acute cardiovascular disease for primary prevention screening identified a high rate of previously undiagnosed cardiovascular risk factors [8].

Medical trainees could learn the necessary family engagement skills through interprofessional interactions and role modeling. Nurses, in particular, are often very skilled at engaging family in care, especially involving families in direct patient care. Practical opportunities for trainees to learn how to engage families in care could include shadowing nurses and allied healthcare professionals (i.e., social workers), rounding with palliative care specialists, communication workshops, and interprofessional simulated learning experiences.

In the outpatient setting, family members often play a key role in reinforcing education and counselling, assisting relatives to express their concerns and medical goals, and improving adherence to treatment. Trainees can incorporate family engagement techniques that are often used in primary care, such as setting the visit agenda to improve communication and ensuring effective information transfer and health literacy through teach-back [9]. Clinicians should encourage family members to accompany their relatives to appointments. During the encounter, clinicians can ask family members to provide additional details about changes in the patient’s status (i.e., decreased functional ability) when relevant. Providing simultaneous lifestyle counselling to the patient and family may be effective at inducing sustainable improvement in modifiable cardiovascular risk factors, such as obesity and smoking cessation.

## Current training in family engagement

Competency-based milestones have been developed by the Accreditation Council for Graduate Medical Education [10]. Each medical specialty has their own specialty-specific milestones and there are some family engagement principles and practices in the recommended curricula. For example, the specialty of Internal Medicine includes a milestone in the Interpersonal and Communication Skills section that involves developing patient- and family-centered communication skills. Another element, history taking, includes accounting for family needs. For the specialty of surgery, the skill of difficult care discussions and patient and family conferences is an advanced competency. Other postgraduate medical education bodies, such as the American College of Cardiology, have produced training recommendations that incorporate family engagement principles into the competency components [11]. Developing cardiologists are expected to [1] “communicate with and educate patients and families across a broad range of cultural, ethnic, and socioeconomic backgrounds”, [2] “interact respectfully with patients, families, and all members of the healthcare team”, [3] “communicate in ways that patients and families can understand the evidence on which recommendations are based,” and [4] “show compassion and effective management of end-of-life issues, including family meetings across the spectrum of patients with heart failure.”

These competencies offer a valuable learning opportunity for trainees on how to effectively engage families in patient care. However, it is uncertain whether they are actually being performed and measured. Training programs should operationalize and evaluate these competency components, as well as consider adding formal instruction and evaluation on other engagement practices, such as meeting family needs and encouraging family presence. Future iterations of the competency-based milestones could integrate additional family engagement practices.

## Challenges

Trainees looking to increase family engagement in their practice may encounter several challenges. Institutional policies may place limits on family visitations, such as a restriction in the number and flexibility of visiting hours. One designated “essential” visitor, particularly for a vulnerable patient, could provide a balance between patient support and safety. Institutional policies may not encourage family presence during sensitive care situations, like invasive procedures, due to infection control reasons, medico-legal considerations, or concern for distraction, although there is little evidence to justify these concerns [12]. There may also be a lack of local resources to provide support to family members. Resistance to a shift in the care approach may also exist at administrative levels. While trainees are typically not in an optimal position to address these systemic issues, early career clinicians can become involved with efforts to advocate for increased family engagement at their institution or on a broader level. Trainees may instead focus their efforts on inviting family to be present, improving communication with family both in person and virtually, being proactive in having goals of care discussions, participating in shared decision making, and promoting efforts for the family member to provide direct care.

Family engagement policies and practices also vary by geographical region and health care system and are likely influenced by societal, cultural, religious, demographic, and socio-economic factors [13]. As a result, there may be differences in the approach to engaging families in care in various settings and populations. For example, the gender role and relationship to the patient (i.e., spouse/partner, son/daughter, friend) could impact caregiver status and desired involvement in care [14]. Trainees may need to receive instruction on cultural sensitivity when caring for individuals from different backgrounds from their own. Thus, there is likely a need to adapt the approach to family engagement instruction based on local considerations.

There is also a need for faculty development approaches for current teachers, so that they can serve as role models for the trainees. Medical schools, universities, and health care systems can offer continuing medical education in the form of training seminars, simulation workshops, and other pedagogical activities to train the teachers. As trainees and early career professionals with increasing proficiency in engaging family in care advance in their careers, they can take leadership roles in implementing family engagement policies, influencing medical school and residency curricula, and training the next generation of learners.

## Conclusion

Engaging family in care involves a shift in the health care approach that has potential benefits to the patient, family, and clinician. Medical training is an appropriate time to learn about and incorporate family engagement techniques into practice. Currently, there are limited training recommendations for engaging families in care from national educational bodies and it is unclear if they are being implemented at all. Training programs should include formal training and evaluation in family engagement practices and trainees should take advantages of the opportunities to engage family throughout the care trajectory.

## Data Availability

Not applicable.
